# Effectiveness of non-pharmacological traditional Chinese medicine combined with conventional therapy in treating fibromyalgia: a systematic review and meta-analysis

**DOI:** 10.3389/fnins.2023.1097475

**Published:** 2023-06-01

**Authors:** Lili Cai, Zhengquan Chen, Juping Liang, Yuanyuan Song, Hong Yu, Jiaye Zhu, Qikai Wu, Xuan Zhou, Qing Du

**Affiliations:** ^1^Department of Rehabilitation, Xinhua Hospital, School of Medicine, Shanghai Jiaotong University, Shanghai, China; ^2^Xinhua Hospital, Shanghai Jiao Tong University School of Medicine, Shanghai, China; ^3^Chongming Hospital, Shanghai University of Medicine and Health Sciences, Shanghai, China

**Keywords:** fibromyalgia, traditional Chinese medicine, pain, depression, quality of life, systematic review

## Abstract

**Objective:**

Fibromyalgia is a chronic musculoskeletal disorder characterized by generalized pain, which is also known as “muscular rheumatism” in Chinese medicine. We undertook this systematic review to evaluate the effectiveness of non-pharmacological traditional Chinese medicine (TCM) combined with conventional therapy on pain, health status, depression, and the quality of life of fibromyalgia patients.

**Methods:**

Studies were retrieved from five electronic databases (PubMed, the Cumulative Index to Nursing and Allied Health, Cochrane Library, Embase, and Web of Science) with publication date up to August 2022. We included randomized controlled trials examining the effects of a combination of non-pharmacological TCM and conventional therapy on pain intensity, health status, depression, and quality of life.

**Results:**

Four randomized controlled trials with 384 fibromyalgia patients met the inclusion criteria. Results of the meta-analysis showed that non-pharmacological TCM combined with conventional therapy exerted significant positive effects on alleviating pain at the post-intervention time point than conventional therapy only (visual analog scale WMD_1_ = −1.410, *P* < 0.01; pressure pain threshold WMD_2_ = 0.830, *P* < 0.001, respectively). Significant differences in pain assessment were also observed between the two groups after a long-term follow-up (12 months) (WMD_1_ = −1.040 and WMD_2_= 0.380, all *P* < 0.05). The combination therapy group also showed a greater reduction in fibromyalgia impact questionnaire than the control group after a long-term follow-up (WMD = −6.690, *P* < 0.05). Depression and pain-related quality of life showed no difference between groups (all *P* > 0.05).

**Conclusion:**

Non-pharmacological TCM combined with conventional therapy may be more effective in alleviating pain and improving health status than conventional therapy only. However, it remains some concerns over the safety and clinic application.

**Systematic review registration:**

Identifier: CRD42022352991.

## 1. Introduction

Fibromyalgia is a chronic musculoskeletal disorder characterized by diffuse pain, fatigue, and sleep disturbances (Clauw, 2014; Arnold et al., 2019). It has been the second most common rheumatologic disorder after osteoarthritis, affecting at least 2 to 4% of the population worldwide (Häuser et al., 2015). The core symptom of fibromyalgia is variable and multifocal pain, which occurs at different intensities and multiple sites during the disease. Individuals with fibromyalgia are more vulnerable to hyperalgesia or allodynia (Chinn et al., 2016), thus leading to cognitive impairment, emotional disorders (depression or anxiety), and somatic symptoms (headaches) (Bair and Krebs, 2020).

Conventional therapy, which is a multidisciplinary and non-invasive treatment, is the preferred choice for fibromyalgia patients according to the guidelines in different countries (Fitzcharles et al., 2013; Macfarlane et al., 2017; Ariani et al., 2021). Conventional therapy includes exercise, education, cognitive behavioral therapy (CBT) and drug therapy. As European League Against Rheumatism (EULAR) revised recommendations suggested (Macfarlane et al., 2017), patient education can be used as a basic treatment, and exercise is a “strong for” recommendation for improving pain and physical function. Psychological therapies, especially CBT, may help to ease pain-related negative emotions, such as depression and anxiety. Besides, as one of the recommended treatments, pharmacotherapy may also be a beneficial supplement for pain and depression relief, which includes gabapentinoids, tricyclic antidepressants (TCAs), and serotonin-norepinephrine reuptake inhibitors (SNRI). These conventional therapies mentioned above recommended by the EULAR revised recommendations are widely recognized and used in clinical practice for fibromyalgia.

Non-pharmacological TCM (traditional Chinese medicine, such as Qigong and acupuncture) is also considered as an effective alternative therapy in managing fibromyalgia recommended by the latest two practice guidelines (Ariani et al., 2021; Qian, 2021). It has been well-accepted among patients with fibromyalgia due to high safety, simple operation, fewer adverse reactions, and no addiction (Deare et al., 2013; Sarzi-Puttini et al., 2020). TCM has obvious superiority in the treatment of chronic pain and concomitant mental disorders as it is based on the concept of wholism and harmony of body and mind (Bushnell et al., 2013; Patel et al., 2020). From the TCM point of view, fibromyalgia is generally classified as “muscular rheumatism”. The pathogenesis includes the internal factor (deficiency of vital Qi) and the external factor (invasion of pathogenic Qi). It leads to the stagnation of blood vessels and loss of muscle nourishment (Yun, 2022a). The negative mood state is a main clinical manifestation of fibromyalgia, and the liver dominates emotions. In order to relieve patients' psychiatric and somatic symptoms, the treatment for fibromyalgia should focus on how to dredge the liver meridian and manage emotions (Yang, 2022; Yun, 2022b).

Although non-pharmacological TCM (such as Qigong and acupuncture) proved to be effective (Sarzi-Puttini et al., 2020), it remains unclear whether a combination of non-pharmacological TCM and conventional therapy is more effective than conventional therapy only in treating fibromyalgia in clinical settings. This systematic review aims to figure out the analgesic effect of integrated TCM and conventional therapy on the health status, depression, and pain-related quality of life of patients with fibromyalgia.

## 2. Methods

The protocol of this systematic review was registered in PROSPERO (No. CRD42022352991) and the work adheres to PRISMA guidelines (Moher et al., 2009).

### 2.1. Search strategy

Systematic searches were conducted in the databases PubMed, the Cumulative Index to Nursing and Allied Health, Cochrane Library, Embase, and Web of Science. We used search terms such as “traditional Chinese medicine”, “TCM”, and “fibromyalgia” to find relevant studies published until August 2022, and the full search strategy was listed in the Supplementary Table 1.

### 2.2. Eligibility criteria

Studies published in English were eligible if they met the following inclusion criteria: (1) Participants: patients over 17 years of age with a diagnosis of fibromyalgia according to the American College of Rheumatology (ACR) criteria or the ACTTION-American Pain Society Pain Taxonomy (AAPT) criteria. (2) Interventions: non-pharmacological TCM combined with conventional therapy. (3) Comparisons: conventional therapy in treating fibromyalgia (such as exercise, psychological therapies, patient education, pharmacotherapy). (4) Outcome measures: Primary outcome measures: Pain intensity: visual analog scale (VAS), and pressure pain threshold (PPT) (Fischer, 1987). Secondary outcome measures: Health status: total Fibromyalgia Impact Questionnaire (FIQ) score (Williams and Arnold, 2011). Depression: Hamilton test score (HAM) and Beck Depression Inventory (BDI) (Beck et al., 1961; Ramos-Brieva and Cordero-Villafafila, 1988). Pain-related quality of life: the bodily pain score of the short form-36 (SF-36). Adverse events: specific adverse events reported during the treatment and follow-up (Bair and Krebs, 2020). Study design: Randomized Controlled Trial (RCT).

Studies were excluded if they were: (1) Self-reported diagnosis without clinical confirmation. (2) Participants in the combination therapy group were treated concomitantly with herbal medicine. (3) Participants in the control group received TCM treatment.

### 2.3. Data extraction

Two reviewers (ZC and LC) independently screened the enrolled articles through title and abstract screening and full-text reading. Extracted data included: source, setting and language, study type, demographics, symptom duration and severity of fibromyalgia, interventions, outcome measures, adverse events, time points, and overall dropout rate. Disagreements were resolved through discussion with a third reviewer (QD).

### 2.4. Risk of bias assessment

The methodological quality of the included studies was evaluated using the Cochrane Risk of Bias Tool 2.0 (Rob 2.0). Grading of Recommendation Assessment, Development, and Evaluation (GRADE) was used to assess the quality of the evidence. JL, YS, and HY performed the quality assessment.

### 2.5. Statistical analysis

Data analysis was performed using Review Manager (RevMan, Version 5.4. Copenhagen: The Nordic Cochrane Center, The Cochrane Collaboration, 2020). A fixed-effects model was used for the quantitative analysis if *I*^2^ ≤ 50%, which represented low to moderate heterogeneity, while a random-effects model was used when *I*^2^ > 50%, to reduce the impact of substantial heterogeneity. The results would be presented as weighted mean difference (WMD) or standardized mean difference (SMD) and 95% confidence interval (CI) with a significance value set as 0.05.

## 3. Results

### 3.1. Identification of studies

**Figure 1:**
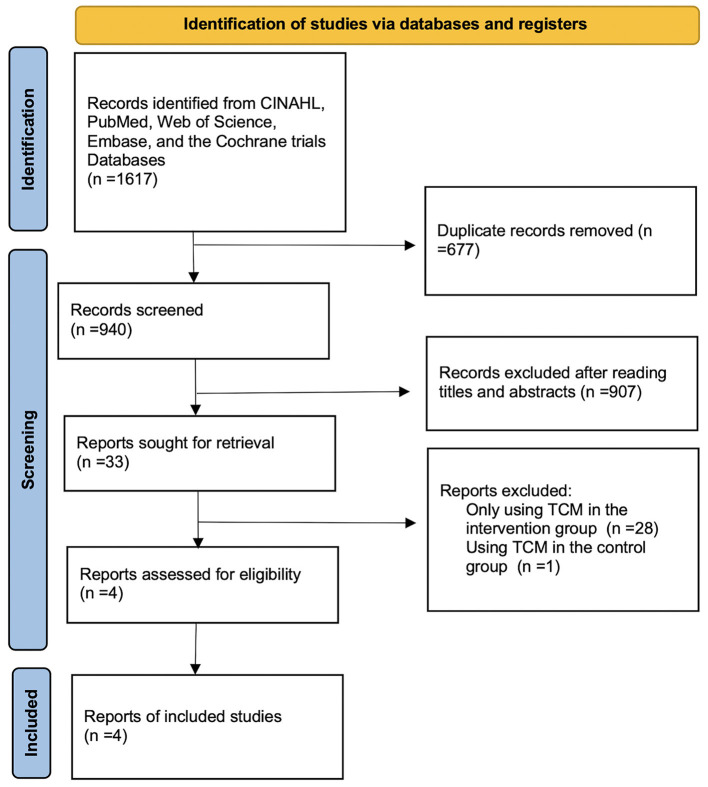
Flowchart of the process of literature search and extraction of studies meeting the inclusion criteria.

The flow diagram of the study selection was provided in Figure 1. Our search retrieved 1617 relevant articles from the five databases. Following the removal of duplicates, the remaining 940 articles were evaluated based on their relevance and publication type. In total, 907 articles with obvious irrelevant topics or non-RCTs were excluded. After the full-text reading, 28 studies only using TCM in the intervention group and 1 study using TCM in the control group were excluded. Four RCTs (Astin et al., 2003; Mannerkorpi and Arndorw, 2004; Targino et al., 2008; Vas et al., 2016) finally met the eligibility criteria.

### 3.2. Studies' characteristics

The baseline characteristics of enrolled patients, a summary of outcome measures, and adverse events in included studies were shown in Table 1. The intervention method, treatment frequency, treatment duration, and follow-up of the combination therapy group or control group were shown in Table 2.

**Table 1:** Characteristics of included studies.

	**Source**	**Setting; language**	**Study type**	**Sample size (% women)**	**Age: mean (SD)**	**Symptom duration and severity**	**Intervention**	**Outcome measures**	**Adverse events (% of intervention group)**	**Time points**	**Overall dropout rate**
1	Astin et al. (2003)	United States; English	RCT	T: 128 (99.2) I: 64 (98.4) C: 64 (100)	T: 47.7 (10.6) yr.	Symptom duration: I: 4.89 (4.15) yr. C: 5.22 (7.31) yr. Severity(FIQ): I: 57.8 (10.8) C: 58.7 (13.5)	Qigong + Mindfulness meditation training	1. Pain: TePsN, Total Myalgic Score 2. Health status: FIQ 3. Quality of life: SF-36 pain score 4. Depression: BDI 5. Function: six minute walk time test 6. Medical care history 7. Coping Strategies Questionnaire	Not provided	I: Baseline 8 weeks 14 weeks 24 weeks C: Baseline 8 weeks 14 weeks 24 weeks	I: 50% C: 48.4% T: 49.2%
2	Mannerkorpi and Arndorw (2004)	Sweden; English	RCT	T: 36 (100) I: 19 (100) C: 17 (100)	T: 45 (8.3) yr.	Symptom duration: 10 (8.5) yr. Severity(FIQ): I: 6.0 (1.8) C: 6.5 (1.9)	Qigong + Body awareness therapy + Medication	1. BARS 2. Health status: FIQ 3. Function: Chair Test and Hand Grip	Increased pain in low back and hips while standing still (54.5)	I: Baseline 3 months C: Baseline 3 months	I: 37% C: 41% T: 38.9%
3	Targino et al. (2008)	Brazil; English	RCT	T: 58 (100) I: 34 (100) C: 24 (100)	I: 52.09 (10.97) yr. C: 51.17 (11.20) yr.	Symptom duration: I: 118.8 (117.3) mo. C: 93.0 (75.25) mo. Severity(VAS): I: 8.0 (4.0-10.0) C: 8.0 (4.0-10.0)	Acupuncture + Tricyclic antidepressant and Exercise	1. Pain: VAS, TePsN, PPT 2. Quality of life: SF-36	Temporary edema (5.8)	I: Baseline 3 months 6 months 12 months 24 months C: Baseline 3 months 6 months 12 months 24 months	I: 5.9% C: 4.2% T: 5.2%
4	Vas et al. (2016)	Spain; English	RCT	T: 162 (100) I: 80 (100) C: 82 (100)	I: 52.3 (9.6) yr. C: 53.2 (9.6) yr.	Symptom duration: I: 70.7 (44.5) mo. C: 69.2 (43.7) mo. Severity(VAS): I: 79.3 (11.0) C: 75.8 (13.3)	Acupuncture + pharmacotherapy	1. Pain: VAS, TePsN, PPT 2. Depression: HAM 3. Health status: FIQ 4. Quality of life: SF-12	Post-acupuncture pain (1.4) Post-acupuncture bruising (2.6) Post-acupuncture vagal symptoms (0.7)	I: Baseline 10 weeks 6 months 12 months C: Baseline 10 weeks 6 months 12 months	I: 8.8% C: 2.5% T: 5.6%

RCT, Randomized Controlled Trial; SD, standard deviation; T, total participants; I, intervention group; C, control group; FIQ, Fibromyalgia Impact Questionnaire; BDI, Beck Depression Inventory; BARS, Body Awareness Rating Scale; HAM, Hamilton test score; VAS, visual analog scale; PPT, pressure pain threshold; TePsN, number of tender points; SF-36, short form 36 questionnaire; SF-12, short form 12 questionnaire.

**Table 2:** Interventions in the included trials.

	**Source**	**Intervention group**	**Control group**	**Duration**	**Follow-up**
1	Astin et al. (2003)	**Qigong:** *Dance of the Phoenix* (a Chinese master). 1h/ time, 1 time/ week. **+** **Mindfulness meditation training:** 2 formal meditation practices (a body scan and sitting meditation). 1.5h/ time, 1 time/ week.	**Education/support:** Short lectures on topics from the book “Your personal guide to living well with fibromyalgia: A handbook for self-care and treatment”. 2.5h/ time, 1 time/ week.	8 weeks	14 weeks 24 weeks
2	Mannerkorpi and Arndorw (2004)	**Qigong:** Relaxation, grounding and concentration. 20minutes/ time, 1 time/ week. **+** **Body awareness therapy:** Breathing and postural techniques. 70minutes/ time, 1 time/ week. **+** **Pharmacotherapy:** Analgesics, anti-depressive medicines or sedatives.	**Normal daily activities without any changes**. **+** **Pharmacotherapy:** Analgesics, anti-depressive medicines or sedatives.	3 months	/
3	Targino et al. (2008)	**Acupuncture:** Ex-HN-3, and LR3, LI4, PC6, GB34, SP6 on both sides. 20 min/ time, 2 times/ week. **+** **Standard care:** Same as the control group.	**Standard care:** 12.5-75mg tricyclic antidepressants. 1 time/ day. 30 min walk + 30 min breathe and mental relaxation exercise. 2 times/ week. Stretching exercises. 2 times/ week.	3 months	6 months 12 months 24 months
4	Vas et al. (2016)	**Individualized Acupuncture**: 20 min/ time, 1 time/ week. **+** **Pharmacotherapy:** Analgesics or antidepressant medication.	**Sham acupuncture:** An acupuncture simulation on the dorsal and lumbar regions by the guide tubes without needles. 20 min/ time, 1 time/ week. **+** **Pharmacotherapy:** Analgesics or antidepressant medication.	10 weeks	6 months 12 months

This review included 4 articles published from 2003 to 2016, and data from 4 studies were included in the meta-analysis. All included studies were RCTs in English. In total, 197 patients were allocated to the combination therapy group and 187 patients received control intervention.

The duration of intervention for fibromyalgia patients ranged from 8 weeks to 3 months, and the frequency of treatment ranged from once to twice a week. Patients in the combination therapy group were treated with non-pharmacological TCM in addition to conventional therapy. Non-pharmacological TCM treatment included Qigong (Astin et al., 2003; Mannerkorpi and Arndorw, 2004) and acupuncture (Targino et al., 2008; Vas et al., 2016). Conventional therapy included mindfulness meditation training (Astin et al., 2003), body awareness therapy plus pharmacotherapy (Mannerkorpi and Arndorw, 2004), tricyclic antidepressants plus exercise (Targino et al., 2008), and conventional pharmacotherapy (Vas et al., 2016).

### 3.3. Primary outcomes

#### 3.3.1. Pain intensity

Two trials assessed pain intensity using VAS and PPT (Targino et al., 2008; Vas et al., 2016). Targino et al. (2008) reported a significant decrease in VAS score and increase in PPT after 3-month and 12-month acupuncture treatment combined with antidepressants and exercise. The same conclusion could be drawn from another article (Vas et al., 2016). The pain intensity reduction was also greater in the acupuncture plus medication group than in the sham acupuncture group after 10-week and 12-month follow-up.

**Figure 2:**
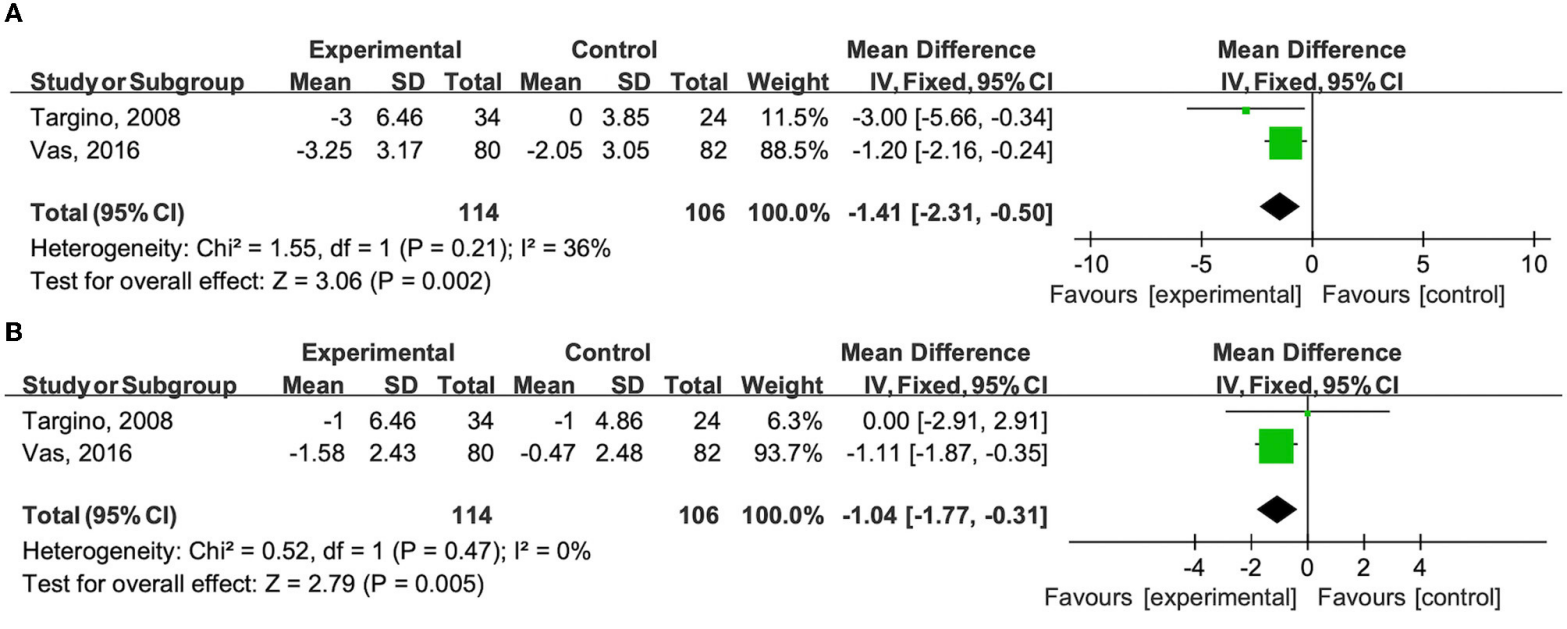
**(A)** Effects of the combination therapy on VAS score in fibromyalgia after the treatment. **(B)** Effects of the combination therapy on VAS score in fibromyalgia after a long-term follow-up.

The pooled data showed that the combination therapy group exhibited a more significant decrease in VAS score than the control group after intervention (WMD −1.410; 95% CI −2.31 to −0.50, *P* < 0.01, *I*^2^ = 36.0%) (Figure 2A) and after a long-term follow-up (WMD −1.040; 95%CI −1.77 to −0.31, *P* < 0.01, *I*^2^ = 0.0%) (Figure 2B).

**Figure 3:**
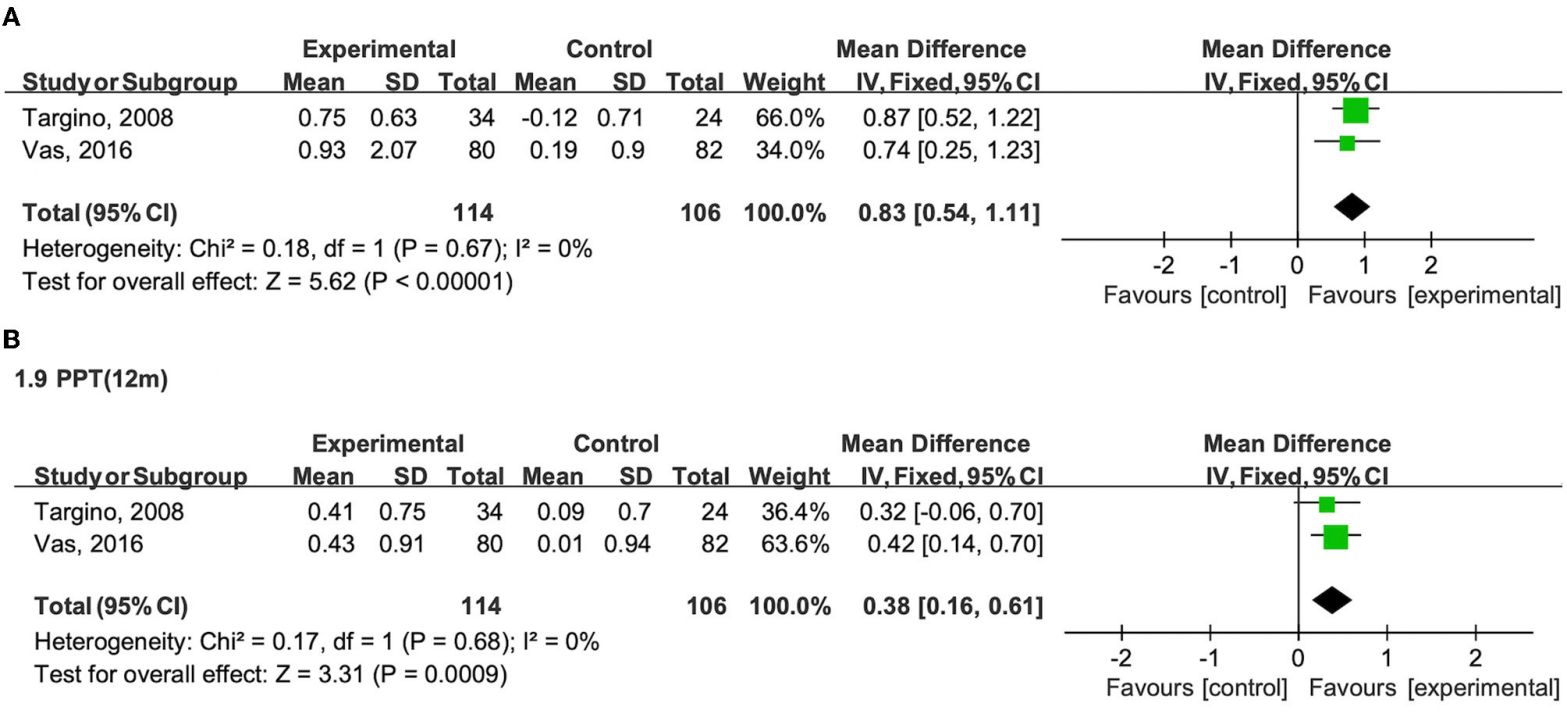
**(A)** Effects of the combination therapy on PPT in fibromyalgia after the treatment. **(B)** Effects of the combination therapy on PPT in fibromyalgia after a long-term follow-up.

PPT was assessed in two studies (Targino et al., 2008; Vas et al., 2016). As shown in Figures 3A, B, patients in the combination therapy group demonstrated a substantially better improvement in PPT than those in the control group after intervention (WMD 0.830; 95% CI 0.54 to 1.11, *P* < 0.001, *I*^2^ = 0.0%) and after a long-term follow-up (WMD 0.380; 95% CI 0.16 to 0.61, *P* < 0.001, *I*^2^ = 0.0%).

### 3.4. Secondary outcomes

#### 3.4.1. Health status

Health status was investigated in 3 studies by using the total FIQ score (Astin et al., 2003; Mannerkorpi and Arndorw, 2004; Vas et al., 2016). FIQ is a 10-item questionnaire to measure the health status of patients with fibromyalgia, with higher scores indicating worse function and symptoms (Burckhardt et al., 1991).

Astin et al. (2003) reported that both Qigong plus mindfulness meditation training group and the control group registered statistically significant improvements after 8-week and 24-week follow-up, but no statistically significant between-group difference for FIQ score was detected. Mannerkorpi and Arndorw (2004) also pointed out that no differences were found between groups after a 3-month follow-up. However, Vas et al. (2016) obtained a result that the FIQ score of the patients in the acupuncture plus medication group was significantly better than in the sham acupuncture group after 10-week and 12-month follow-up.

**Figure 4:**
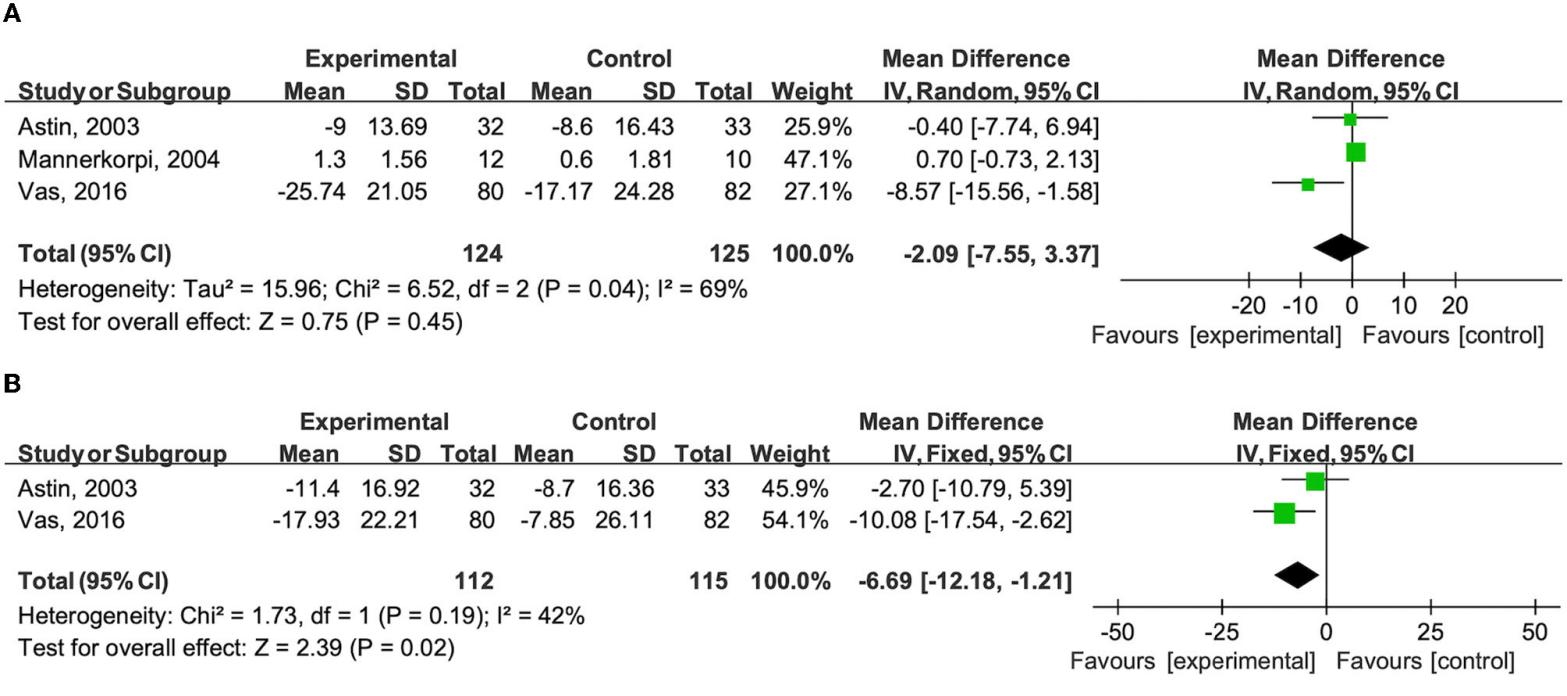
**(A)** Effects of the combination therapy on total FIQ score in fibromyalgia after the treatment. **(B)** Effects of the combination therapy on total FIQ score in fibromyalgia after a long-term follow-up.

When the results were pooled, no significant differences were shown between the combination therapy group and control group after intervention (WMD −2.090; 95%CI −7.55 to 3.37, *P* = 0.450, *I*^2^ = 69.0%) (Figure 4A). However, the pooled results showed a greater reduction in total FIQ score than the control group after a long-term follow-up (WMD −6.690; 95%CI −12.18 to −1.21, *P* < 0.05, *I*^2^ = 42.0%) (Figure 4B).

After removing the study from Vas et al. (2016) based on sensitivity analysis, there was no significant statistical heterogeneity and no significant results were found after intervention (WMD 0.66; 95% CI −0.74 to 2.06, *P* = 0.36, *I*^2^ = 0%).

#### 3.4.2. Depression

Regarding the assessment of depression, Astin et al. (2003) used BDI while Vas et al. (2016) measured HAM. Patients with higher scores of BDI and HAM may have more severe depression.

The study from Vas et al. (2016) showed that HAM scores decreased in the acupuncture plus medication group and control group after the 10-week and 6-month treatments, but the difference was not statistically and clinically significant. Astin et al. (2003) also yielded no positive results between Qigong + mindfulness meditation training group and the control group after 8-week and 24-week follow-up.

Compared to the control group, no significant improvement in the combination therapy group was observed in the level of depression after intervention (SMD −0.090; 95% CI −0.35 to 0.17, *P* = 0.500, *I*^2^ = 0.0%) (Supplementary Figure 1A). The results led to a similar conclusion after a long-term follow-up (SMD −0.150; 95%CI −0.41 to 0.11, *P* = 0.260, *I*^2^ = 0.0%) (Supplementary Figure 1B).

#### 3.4.3. Pain-related quality of life

Two studies reported pain-related quality of life using the bodily pain score of the SF-36 (Astin et al., 2003; Targino et al., 2008).

Compared to the control group, the pooled data showed no more improvement in the bodily pain score of the SF-36 in the combination therapy group after intervention (WMD −0.050; 95%CI −7.84 to 7.74, *P* = 0.990, *I*^2^ = 39.0%) and after a long-term follow-up (WMD −0.890; 95%CI −9.84 to 8.06, *P* = 0.850, *I*^2^ = 0.0%). The results are presented in Supplementary Figures 2A, B.

#### 3.4.4. Adverse events

Three studies mentioned adverse events (Mannerkorpi and Arndorw, 2004; Targino et al., 2008; Vas et al., 2016). Over half of the patients with fibromyalgia in the Qigong group reported increased pain in low back and hips while standing still (Mannerkorpi and Arndorw, 2004). With regard to the adverse events of acupuncture in 2 trials (Targino et al., 2008; Vas et al., 2016), 5.8% of patients with fibromyalgia in the combination therapy group reported temporary oedema, 1.4% reported post-acupuncture pain, 2.6% reported post-acupuncture bruising, and 0.7% reported post-acupuncture vagal symptoms.

### 3.5. Quality appraisal

The risk of bias assessment is provided in the Supplementary material (Supplementary Figure 3). Two studies had a high risk of bias due to no blinding of participants or personnel and a high dropout rate (Astin et al., 2003; Mannerkorpi and Arndorw, 2004). The other two studies were respectively classified as moderate and low risk of bias (Table 3) (Targino et al., 2008; Vas et al., 2016). The quality of the evidence for each outcome measure was rated from moderate to very low. Outcomes including VAS and PPT showed moderate quality, while the remaining six outcome measures showed very low quality (Supplementary Table 2).

**Table 3:** Risk of bias assessment of the included randomized controlled trials.

	**Article, Year**	**Randomization Process**	**Deviations from the intended interventions**	**Missing outcome data**	**Measurement of the outcome**	**Selection of the reported result**	**Overall bias**
1	Astin et al. (2003)	Y/Y/N	Y/Y/N/NA/NA/N/Y	N/PN/PN/NA	N/N/N/NA/NA	NI/NI/NI	High
		Low	High	Low	Low	Some concerns	
2	Mannerkorpi and Arndorw (2004)	NI/NI/N	Y/Y/N/NA/NA/N/ Y	N/PN/PN/NA	N/N/N/NA/NA	NI/NI/NI	High
		Some concerns	High	Low	Low	Some concerns	
3	Targino et al. (2008)	Y/Y/N	Y/Y/N/NA/NA/Y/NA	Y/NA/NA/NA	N/N/N/NA/NA	NI/NI/NI	Some concerns
		Low	Low	Low	Low	Some concerns	
4	Vas et al. (2016)	Y/Y/N	PN/Y/N/NA/NA/Y/NA	Y/NA/NA/NA	N/N/N/NA/NA	Y/N/N	Low
		Low	Low	Low	Low	Low	

Y, Yes; N, No; PY, Probably Yes; PN, Probably No; NI, No Information; NA, Not Applicable.

## 4. Discussion

This meta-analysis evaluated the effect of a combination of non-pharmacological TCM and conventional therapy in treating fibromyalgia compared to conventional therapy only. The present meta-analysis of data from 4 RCTs revealed that non-pharmacological TCM combined with conventional therapy was superior to the control intervention in the improvement of pain and health status.

Fibromyalgia is characterized by poor circulation of Qi and blood that could not nourish muscles in the perspective of TCM. The moody state is a prominent external manifestation of this disease, associated with physical dysfunction such as pain and sleep quality. Therefore, focusing on the movement of Qi, mainly in the liver meridian, may be the key to improving the moody state and somatic dysfunctions (Yang, 2022; Yun, 2022b). Blood stasis leads to inadequate blood flow to the liver and stagnation of liver Qi, which hinders the liver meridian from timely regulation of other organs. It may result in various somatic dysfunctions, especially pain. In TCM intervention, the key to alleviating pain is to disperse stagnated liver Qi and eliminate Qi stagnation.

Central sensitization marked by the dysfunction of neuro-circuits is one of the main pathogenesis of fibromyalgia (Siracusa et al., 2021). Central sensitization refers to the enhanced transmission of nociceptive signals in the dorsal horn of the spinal cord. Animal studies showed that acupuncture may activate bioactive chemicals (such as opioids) and inhibit the activation of spinal microglia to modulate the local inflammatory environment to inhibit central sensitization (Lai et al., 2019). In addition, the pathogenesis of fibromyalgia may also link to inflammatory factors (Littlejohn and Guymer, 2018). Moderate-intensity exercise has been proven to decrease fibromyalgia patients' blood levels of cytokines (Bote et al., 2013). Qigong is a moderate-intensity exercise incorporating Qi regulation and supporting the righteous Qi (Jiao et al., 2019), as well regulating breath control and mental adjustment. It may also ameliorate symptoms (especially pain) caused by liver Qi stagnation through physical and emotional regulation in the process of training (Yeung et al., 2018). Meanwhile, clinical practice and the animal experiment proved that stimulating Yintang (Ex-HN-3) may be related to inflammatory pathways, and it is considered effective in improving negative emotions (Armour et al., 2019). Taichong (LR3) and Yanglingquan (GB34) are also classical acupoints employed (WHO, 1991; Qiao et al., 2020).

Our results showed that a significantly more decrease in pain was found in the combination therapy group than that in the control group. However, it seems difficult to define a minimal clinical important difference (MCID) in pain improvement in patients with fibromyalgia. A consensus statement indicated that a 1.0 cm reduction in the 10 cm VAS may represent a “minimal” or “little” change in chronic pain, while a 2.0 to 2.7 cm reduction may be more clinically meaningful (Dworkin et al., 2008). The decrease of VAS scores (WMD = −1.410) in this meta-analysis suggests a “little” difference in pain between the combination therapy group and the control group. Since MCID values varied widely depending on the type of chronic pain and baseline pain intensity (Muñoz-Leyva and Chan, 2020), future studies should focus on the calculation of MCID in pain measurements (e.g., VAS and PPT) in patients with fibromyalgia.

There were no significant differences in depression and pain-related quality of life between the combination therapy group and the control group. A likely explanation is that TCM emphasizes individualized treatment, however, none of the included studies mentioned TCM diagnosis (Mist et al., 2011). Acupuncture points and the frequency or intensity of interventions may focus only on core symptoms such as pain, while the individual needs such as quality of life and improving depression are possibly ignored. Another underlying reason is that though Qigong is effective in regulating body and mind, the exercise requires continuous participation, long-term adherence, and high-level cognitive ability for the patients (Jones and Liptan, 2009; Sawynok and Lynch, 2017). No results showed an improvement in depression and quality of life due to the high dropout rate (37–50%). Moreover, patients with comorbid depression can benefit from specific treatment such as psychopharmacological treatment or cognitive behavioral therapies recommended by the guidelines, but only one article mentioned tricyclic antidepressants were used and no articles included in our systematic review employed CBT as conventional therapy.

Three studies mentioned the adverse events in the combination therapy group, including temporary edema (acupuncture), post-acupuncture pain, post-acupuncture bruising, post-acupuncture vagal symptoms, and increased pain in low back and hips in Qigong exercise (Mannerkorpi and Arndorw, 2004; Targino et al., 2008; Vas et al., 2016). Acupuncture is an invasive treatment, but bleeding and swelling after acupuncture can be relieved by a cold compress. It is necessary to inform patients of the underlying risks before administering needles and apply acupuncture more gently to minimize side effects. It is worth noting that up to 54.5% of patients who took part in Qigong plus body awareness therapy reported increased pain in low back and hips while standing still (Mannerkorpi and Arndorw, 2004). The possible explanation may be that Qigong is a moderate-intensity exercise that may increase the pain and discomfort of patients with fibromyalgia due to exercise-induced fatigue (Lima et al., 2017). Hence, we need to pay more attention to patients and require timely feedback on any discomfort during Qigong intervention. Additionally, to reduce the rate of side effects and increase patient compliance, we should appropriately increase the frequency of rest between training sessions, and guide patients on how to perform relaxation and stretching after training (Garber et al., 2011).

## 5. Limitations

Some limitations need to be addressed when interpreting the results. First, it may be difficult to draw any sound conclusion because only four eligible RCTs were included in the meta-analysis. Different types of interventions (Qigong, acupuncture, exercise, etc.) and acupoints (standardized or individualized acupuncture) were employed in these studies. The high methodological heterogeneity may restrict the possibility of inferences from the present results and subgroup analysis could not be conducted due to the limited number of studies. A standardized intervention protocol should be developed in the future. Second, two of the included studies showed a high risk of bias due to a high overall dropout rate. Improving the acceptance and comfort of TCM intervention may be a feasible plan to reduce the dropout rate. Future studies should focus on stretching and relaxation after exercise to reduce pain. Online guidance or supervision may be a key way to enhance the adherence of fibromyalgia patients to TCM. Third, although there was a more significant decrease in pain intensity in the combination therapy group than in the control group, it remained unclear whether the effect size reached MCID. Hence the effect of combination therapy on relieving pain should be interpreted with caution and the calculation of MCID in pain measurements warrants further research.

## 6. Conclusion

Non-pharmacological TCM combined with conventional therapy may be more effective than conventional therapy only in alleviating pain and improving the health status of patients with fibromyalgia. A long-term effect of non-pharmacological TCM combined with conventional therapy was also found in the treatment of pain and health status. However, our results should be applied to the clinic with caution due to the limited number of included studies, methodological heterogeneity, and potential adverse events. To increase the safety and adherence to non-pharmacological TCM, future studies should add stretching and relaxation after exercise and provide online supervision to fibromyalgia patients during the intervention.

## Data availability statement

The original contributions presented in the study are included in the article/Supplementary material, further inquiries can be directed to the corresponding authors.

## Author contributions

LC and ZC conceived the idea and contributed to the writing of the manuscript and screened the enrolled articles and completed the data extraction. LC, JZ, and QW performed the literature search. JL, YS, and HY assessed the risk of bias and the quality of the evidence. LC, ZC, and JL conducted the statistical analyses and interpreted the results. QD and XZ revised the manuscript and gave guidance throughout the process of this study. All authors read and approved the summited and final version of the manuscript to be published.
