# Supplementary material for: Effectiveness of non-pharmacological traditional Chinese medicine combined with conventional therapy in treating fibromyalgia: a systematic review and meta-analysis
Source: Front Neurosci. 2023 Jun 1;17:1097475. doi: 10.3389/fnins.2023.1097475 (PMC10267337; doi:10.3389/fnins.2023.1097475)
Supplement: Supplementary file 1 [file Data_Sheet_1.docx]

Supplementary Material

# Supplementary Tables

## Supplementary Table 1. Search strategy

| Search strategy | |
| --- | --- |
| Pubmed: (n=188) August 19, 2022 | #1 “Single-Blind Method”[Mesh] OR “Double-Blind Method”[Mesh] OR “Randomized Controlled Trials As Topic”[Mesh] OR “Intention To Treat Analysis”[Mesh] OR “Controlled Clinical Trials As Topic”[Mesh] OR “Clinical Trials As Topic”[Mesh] OR “Clinical Trial” [Publication Type] OR “Randomized Controlled Trial” [Publication Type]  #2 “Random*”[Text Word] OR “Allocation”[Text Word] OR “Random Allocation”[Text Word] OR “Placebo”[Text Word] OR “Single Blind”[Text Word] OR “Double Blind”[Text Word] OR “Randomized Controlled Trial*”[Text Word] OR “RCT”[Text Word]  #3 #1 OR #2  #4 Animals NOT Humans  #5 #3 NOT #4  #6 “Fibromyalgia”[Mesh] OR “Fibromyalgia*”[Title/Abstract] OR “Fibromyositis”[Title/Abstract]  #7 “Medicine, Chinese traditional”[MeSH] OR “Chinese medicine”[Title/Abstract] OR “Traditional medicine”[Title/Abstract] OR “Acupuncture therapy”[MeSH] OR “Acupuncture”[MeSH] OR “Acupuncture”[Title/Abstract] OR “Electroacupuncture”[Title/Abstract] OR “Percussopunctator”[Title/Abstract] OR “Acupotom*”[Title/Abstract] OR “Needles”[MeSH] OR “Needle*”[Title/Abstract] OR “Needling”[Title/Abstract] OR “Moxibustion”[Title/Abstract] OR “Meridian*”[Title/Abstract] OR “Acupoint*”[Title/Abstract] OR “Tai ji”[MeSH] OR “Tai ji” [Title/Abstract] OR “Tai chi”[Title/Abstract] OR “Taiji*”[Title/Abstract] OR “Taichi*”[Title/Abstract] OR “Qigong”[MeSH] OR “Qigong”[Title/Abstract] OR “Qi gong”[Title/Abstract] OR “Baduanjin”[Title/Abstract] OR “Liuzijue”[Title/Abstract] OR “Liu zi jue”[Title/Abstract] OR “Wuqinxi”[Title/Abstract] OR “Wu qin xi”[Title/Abstract] OR “Yijinjing”[Title/Abstract] OR “Yi jin jing”[Title/Abstract] OR “Chinese massage”[Title/Abstract] OR “Tuina”[Title/Abstract] OR “Tui na”[Title/Abstract] OR “Manipulation”[Title/Abstract] OR “Cupping therapy” [MeSH] OR “Cupping”[Title/Abstract]  #8 #5 AND #6 AND #7 |
| Cochrane Library: (n=300) August 19, 2022 | #1 ‘parallel’:ti,ab,kw OR ‘observational’:ti,ab,kw OR ‘cross-sectional’:ti,ab,kw OR ‘pre-post’:ti,ab,kw OR ‘before-after’:ti,ab,kw OR ‘controlled trial*’:ti,ab,kw OR ‘random*’:ti,ab,kw OR ‘intervention*’:ti,ab,kw  #2 ‘trial’:pt  #3 #1 OR #2  #4 MeSH descriptor: [fibromyalgia] explode all trees  #5 ‘fibromyalgia*’:ti,ab,kw OR ‘fibromyositis’:ti,ab,kw  #6 #4 OR #5  #7 MeSH descriptor: [medicine, Chinese traditional] explode all trees  #8 MeSH descriptor: [acupuncture] explode all trees  #9 MeSH descriptor: [acupuncture therapy] explode all trees  #10 MeSH descriptor: [needles] explode all trees  #11 MeSH descriptor: [tai ji] explode all trees  #12 MeSH descriptor: [qigong] explode all trees  #13 MeSH descriptor: [cupping therapy] explode all trees  #14 ‘Chinese medicine’:ti,ab,kw OR ‘traditional medicine’:ti,ab,kw OR ‘acupuncture’:ti,ab,kw OR ‘electroacupuncture’:ti,ab,kw OR ‘percussopunctator’:ti,ab,kw OR ‘acupotom*’:ti,ab,kw OR ‘needle*’:ti,ab,kw OR ‘needling’:ti,ab,kw OR ‘moxibustion’:ti,ab,kw OR ‘meridian*’:ti,ab,kw OR ‘acupoint*’:ti,ab,kw OR ‘tai ji’:ti,ab,kw OR ‘tai chi’:ti,ab,kw OR ‘taiji*’:ti,ab,kw OR ‘taichi*’:ti,ab,kw OR ‘qigong’:ti,ab,kw OR ‘qi gong’:ti,ab,kw OR ‘baduanjin’:ti,ab,kw OR ‘liuzijue’:ti,ab,kw OR ‘liu zi jue’:ti,ab,kw OR ‘wuqinxi’:ti,ab,kw OR ‘wu qin xi’:ti,ab,kw OR ‘yijinjing’:ti,ab,kw OR ‘yi jin jing’:ti,ab,kw OR ‘Chinese massage’:ti,ab,kw OR ‘tuina’:ti,ab,kw OR ‘tui na’:ti,ab,kw OR ‘manipulation’:ti,ab,kw OR ‘cupping’:ti,ab,kw  #15 #7 OR #8 OR #9 OR #10 OR #11 OR #12 OR #13 OR #14  #16 #3 AND #6 AND #15 |
| Embase: (n=569) August 19, 2022 | #1 ‘parallel’/exp OR ‘observational’/exp OR ‘cross-sectional’/exp OR ‘pre-post’/exp OR ‘before-after’/exp OR ‘controlled trial*’/exp OR ‘random*’/exp OR ‘intervention*’/exp  #2 ‘parallel’:ab,ti OR ‘observational’:ab,ti OR ‘cross-sectional’:ab,ti OR ‘pre-post’:ab,ti OR ‘before-after’:ab,ti OR ‘controlled trial*’:ab,ti OR ‘random*’:ab,ti OR ‘intervention*’:ab,ti  #3 #1 OR #2  #4 ‘fibromyalgia’/exp OR ‘fibromyalgia*’:ab,ti OR ‘fibromyositis’:ab,ti  #5 ‘traditional medicine’/exp OR ‘traditional medicine’:ab,ti OR ‘Chinese medicine’:ab,ti OR ‘acupuncture’/exp OR ‘acupuncture’:ab,ti OR ‘electroacupuncture’:ab,ti OR ‘percussopunctator’:ab,ti OR ‘acupotom*’:ab,ti OR ‘needle’/exp OR ‘needle*’:ab,ti OR ‘needling’:ab,ti OR ‘moxibustion’/exp OR ‘moxibustion’:ab,ti OR ‘meridian*’:ab,ti OR ‘acupoint*’:ab,ti OR ‘tai chi’/exp OR ‘tai chi’:ab,ti OR ‘tai ji’:ab,ti OR ‘taiji*’:ab,ti OR ‘taichi*’:ab,ti OR ‘qigong’/exp OR ‘qigong’:ab,ti OR ‘qi gong’:ab,ti OR ‘baduanjin’:ab,ti OR ‘liuzijue’:ab,ti OR ‘liu zi jue’:ab,ti OR ‘wuqinxi’:ab,ti OR ‘wu qin xi’:ab,ti OR ‘yijinjing’:ab,ti OR ‘yi jin jing’:ab,ti OR ‘Chinese massage’:ab,ti OR ‘tuina’:ab,ti OR ‘tui na’:ab,ti OR ‘manipulation’:ab,ti OR ‘cupping’:ab,ti  #6 #3 AND #4 AND #5 |
| Web of Science: (n=308) August 19, 2022 | #1 TS=(parallel OR observational OR cross-sectional OR pre-post OR before-after OR ‘controlled trial*’ OR random* OR intervention*)  #2 TS=(fibromyalgia OR fibromyalgia* OR fibromyositis)  #3 TS=(‘traditional medicine’ OR ‘Chinese medicine’ OR acupuncture OR electroacupuncture OR percussopunctator OR acupotom* OR needle* OR needling OR moxibustion OR meridian* OR acupoint* OR ‘tai ji’ OR ‘tai chi’ OR taiji* OR taichi* OR qigong OR ‘qi gong’ OR “tai ji quan” OR “tai chi quan” OR baduanjin OR liuzijue OR ‘liu zi jue’ OR wuqinxi OR ‘wu qin xi’ OR yijinjing OR ‘yi jin jing’ OR ‘Chinese massage’ OR tuina OR ‘tui na’ OR manipulation OR cupping)  #4 #1 AND #2 AND #3 |
| CINAHL: (n=252) August 19, 2022 | S1 MH(“random assignment” OR “placebos” OR “placebo effect” OR “single-blind studies” OR “double-blind studies” OR “triple-blind studies” OR “randomized controlled trials” OR “comparative studies” OR “evaluation research” OR “prospective studies” OR “crossover design” OR “clinical trials” OR “clinical trial registry”)  S2 TX(“random*” OR “allocation” OR “random allocation” OR “placebo*” OR “randomi?ed controlled trial*” OR “clinical trial*” OR “comparative stud*” OR “evaluation stud*” OR “follow-up stud*” OR “prospective stud*” OR “crossover stud*” OR “control*” OR “prospective*” OR “volunteer*” OR “RCT”) OR TX((“singl*” OR “doubl*” OR “trebl*” OR “tripl*”) AND (“mask*” OR “blind*”))  S3 PT(“randomized controlled trial” OR “clinical trial*”)  S4 S1 OR S2 OR S3  S5 MH(“fibromyalgia”) OR AB(“fibromyalgia*” OR “fibromyositis” )  S6 MH(“medicine, Chinese traditional+” OR “needles” OR “tai chi” OR “cupping therapy”) OR AB (“Chinese medicine” OR “traditional medicine” OR “acupuncture” OR “electroacupuncture” OR “percussopunctator” OR “acupotom*” OR “needle*” OR “needling” OR “moxibustion” OR “meridian*” OR “acupoint*” OR “tai ji” OR “tai chi” OR “taiji*” OR “taichi*” OR “qigong” OR “qi gong” OR “baduanjin” OR “liuzijue” OR “liu zi jue” OR “wuqinxi” OR “wu qin xi” OR “yijinjing” OR “yi jin jing” OR “Chinese massage” OR “tuina” OR “tui na” OR “manipulation” OR “cupping”)  S6 S4 AND S5 AND S6 |

## Supplementary Table 2. Quality of the evidence

| **Efficacy of A Combination of Non-pharmacological Traditional Chinese Medicine and Conventional Therapy in Treating Fibromyalgia: A Systemic Review and Meta-Analysis** | | | | | | |
| --- | --- | --- | --- | --- | --- | --- |
| **Patient or population:** patients with fibromyalgia **Settings:**  **Intervention:** Non-pharmacological TCM plus conventional therapy | | | | | | |
| **Outcomes** | **Illustrative comparative risks* (95% CI)** | | **Relative effect (95% CI)** | **No of Participants (studies)** | **Quality of the evidence (GRADE)** | **Comments** |
|  | Assumed risk | Corresponding risk |  |  |  |  |
|  | **Control** | Non-pharmacological TCM plus conventional therapy |  |  |  |  |
| **VAS(after intervention)** |  | The mean vas(after intervention) in the intervention groups was **1.41 lower** (2.31 to 0.5 lower) |  | 220 (2 studies) | ⊕⊕⊕⊝ **moderate**^1^ |  |
| **VAS(long-term follow-up)** |  | The mean vas(long-term follow-up) in the intervention groups was **1.04 lower** (1.77 to 0.31 lower) |  | 220 (2 studies) | ⊕⊕⊕⊝ **moderate**^1^ |  |
| **PPT(after intervention)** |  | The mean ppt(after intervention) in the intervention groups was **0.83 higher** (0.54 to 1.11 higher) |  | 220 (2 studies) | ⊕⊕⊕⊝ **moderate**^1^ |  |
| **PPT(long-term follow-up)** |  | The mean ppt(long-term follow-up) in the intervention groups was **0.38 higher** (0.16 to 0.61 higher) |  | 220 (2 studies) | ⊕⊕⊕⊝ **moderate**^1^ |  |
| **life quality(after intervention)** |  | The mean life quality(after intervention) in the intervention groups was **0.05 lower** (7.84 lower to 7.74 higher) |  | 123 (2 studies) | ⊕⊝⊝⊝ **very low**^1,3^ |  |
| **life quality(long-term follow-up)** |  | The mean life quality(long-term follow-up) in the intervention groups was **0.89 lower** (9.84 lower to 8.06 higher) |  | 123 (2 studies) | ⊕⊝⊝⊝ **very low**^1^ |  |
| **depression(after intervention)** |  | The mean depression(after intervention) in the intervention groups was **0.09 standard deviations lower** (0.35 lower to 0.17 higher) |  | 226 (2 studies) | ⊕⊝⊝⊝ **very low**^1,3^ | Standardized Mean Difference |
| **depression(long-term follow-up)** |  | The mean depression(long-term follow-up) in the intervention groups was **0.15 standard deviations lower** (0.41 lower to 0.11 higher) |  | 226 (2 studies) | ⊕⊝⊝⊝ **very low**^1,3^ | Standardized Mean Difference |
| **FIQ(after intervention)** |  | The mean fiq(after intervention) in the intervention groups was **2.09 lower** (7.55 lower to 3.37 higher) |  | 249 (3 studies) | ⊕⊝⊝⊝ **very low**^1,2,3^ |  |
| **FIQ(long-term follow-up)** |  | The mean fiq(long-term follow-up) in the intervention groups was **6.69 lower** (12.18 to 1.21 lower) |  | 227 (2 studies) | ⊕⊝⊝⊝ **very low**^1,3^ |  |
| *The basis for the **assumed risk** (e.g. the median control group risk across studies) is provided in footnotes. The **corresponding risk** (and its 95% confidence interval) is based on the assumed risk in the comparison group and the **relative effect** of the intervention (and its 95% CI).  **CI:** Confidence interval; | | | | | | |
| GRADE Working Group grades of evidence **High quality:** Further research is very unlikely to change our confidence in the estimate of effect.  **Moderate quality:** Further research is likely to have an important impact on our confidence in the estimate of effect and may change the estimate. **Low quality:** Further research is very likely to have an important impact on our confidence in the estimate of effect and is likely to change the estimate. **Very low quality:** We are very uncertain about the estimate. | | | | | | |
| ^1^ Total population size is less than 400. ^2^ I2 > 50%. ^3^ >25% of the participants was from studies with high risk of bias. | | | | | | |

# Supplementary Figures

#
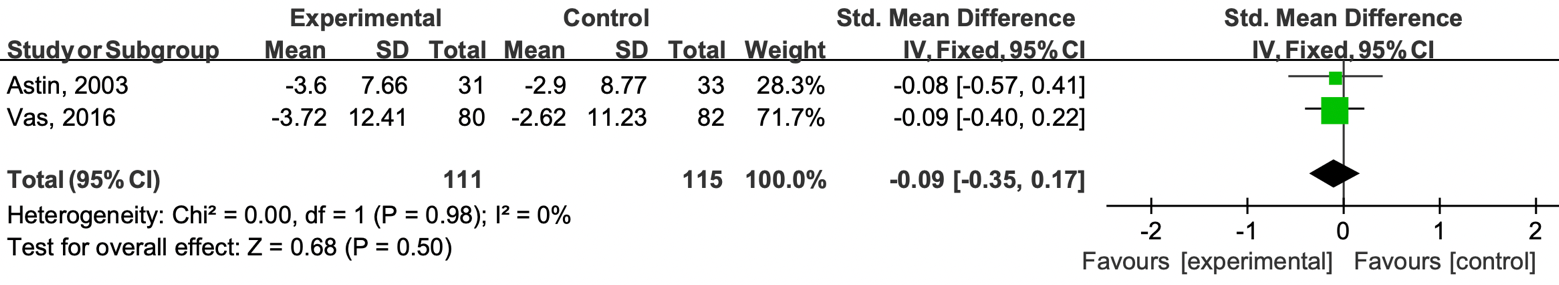


## Supplementary Figure 1A. Effects of the combination therapy on depression in fibromyalgia after the treatment.


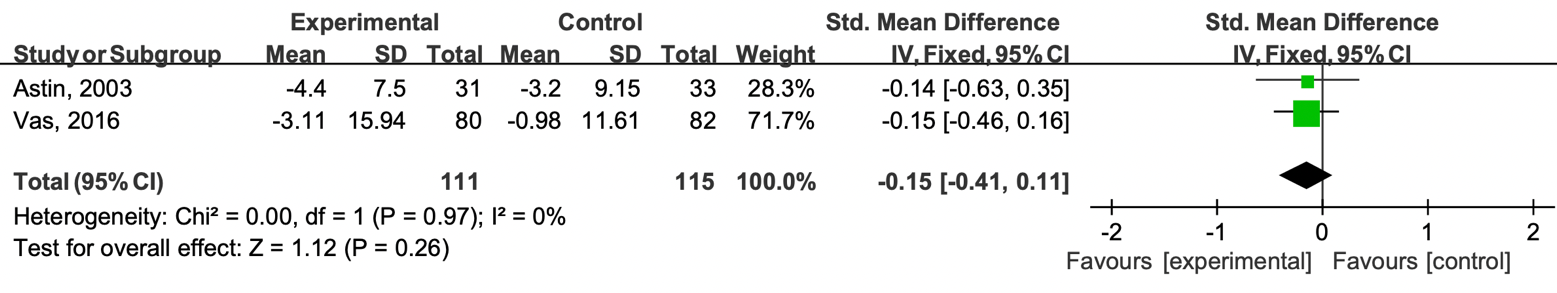


## Supplementary Figure 1B. Effects of the combination therapy on depression in fibromyalgia after a long-term follow-up.


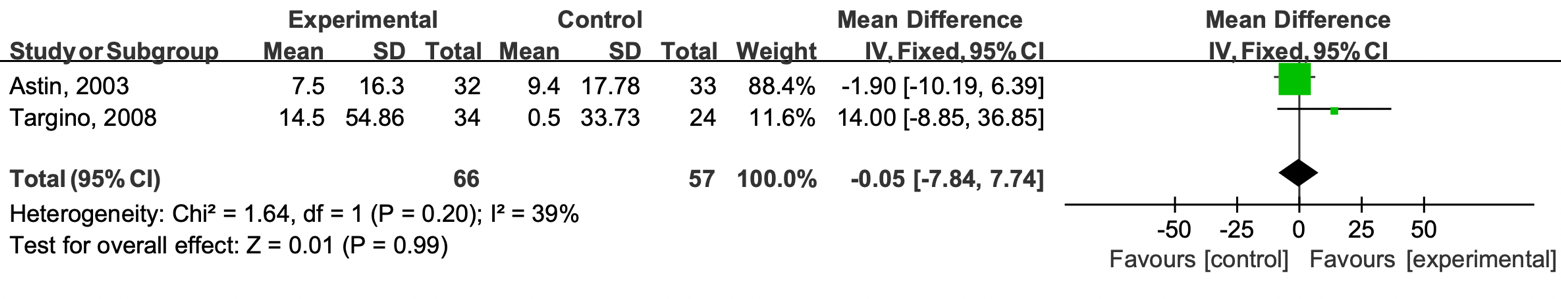


## Supplementary Figure 2A. Effects of the combination therapy on quality of life in fibromyalgia after the treatment.

##
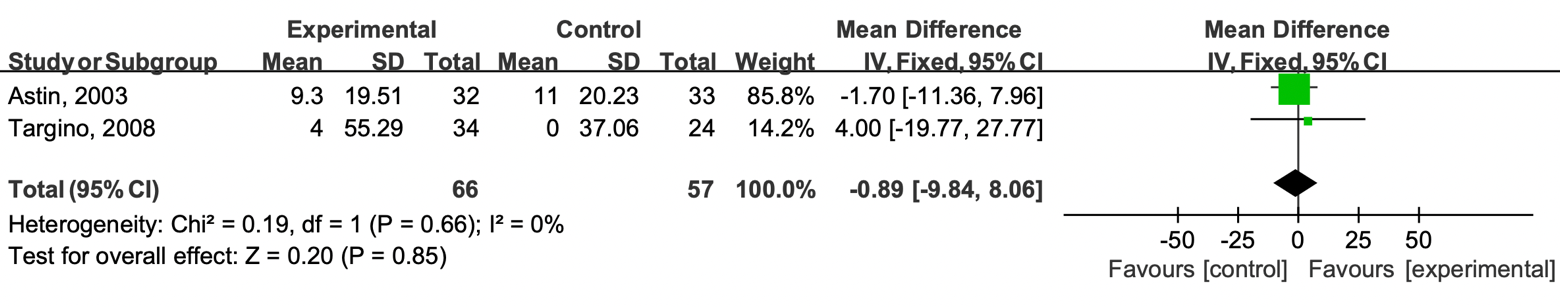


## Supplementary Figure 2B. Effects of the combination therapy on quality of life in fibromyalgia after a long-term follow-up.


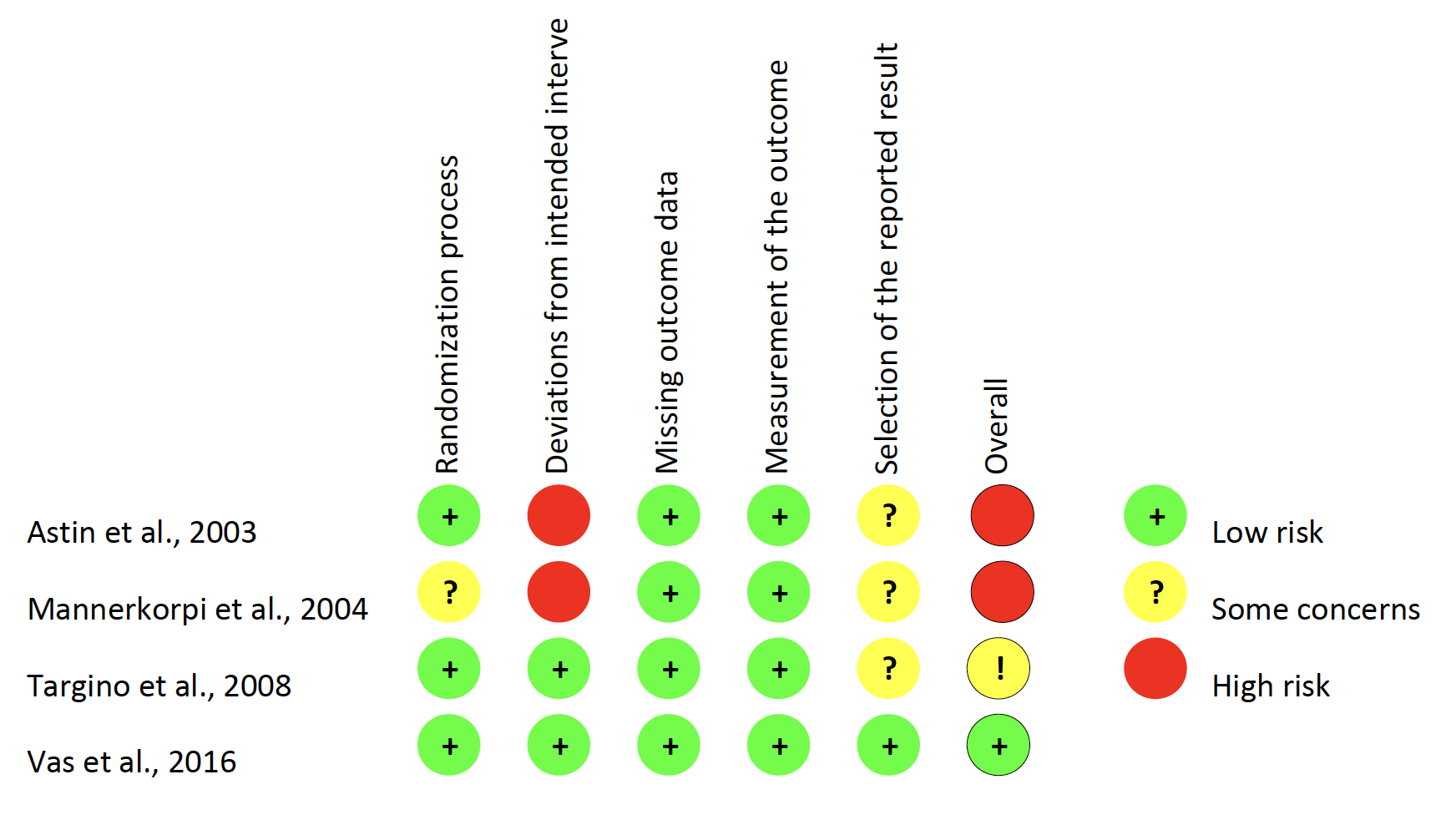


## Supplementary Figure 3. Risk of bias of included studies for meta-analysis.
