# Supplementary material for: Effectiveness of non-pharmacological traditional Chinese medicine combined with conventional therapy in treating fibromyalgia: a systematic review and meta-analysis
Source: Front Neurosci. 2023 Jun 1;17:1097475. doi: 10.3389/fnins.2023.1097475 (PMC10267337; doi:10.3389/fnins.2023.1097475)
Supplement: Supplementary file 2 [file Table_2.DOCX]

**Title Page**

**Title**

Effectiveness of Non-pharmacological Traditional Chinese Medicine Combined with Conventional Therapy in Treating Fibromyalgia: A Systematic Review and Meta-Analysis

**Authors**

Lili Cai^1,3†^ (First author), B.M.

Zhengquan Chen^1†^ (Co-first author), M.S.

Juping Liang^1†^ (Co-first author), M.S.

Yuanyuan Song^2^ (Co-author), B.S.

Hong Yu^1^ (Co-author), B.S.

Jiaye Zhu^1^ (Co-author), B.S.

Qikai Wu^1^ (Co-author), M.S.

Xuan Zhou^1*^ (Co-corresponding author), M.M.

Qing Du^1,2*^ (Corresponding author), Ph.D.

^*^ Corresponding authors.

^†^. These authors contributed equally to this work.

1. Department of Rehabilitation, Xinhua Hospital, School of Medicine, Shanghai Jiaotong University, Shanghai, China

2. Chongming Hospital, Shanghai University of Medicine & Health Sciences, Shanghai, China

3. Xinhua Hospital, Shanghai Jiao Tong University School of Medicine, Shanghai, 200092, China

**Disclosure**

The authors have nothing to disclose.

**Corresponding author
*Name:*** Qing Du

***Address:*** Department of Rehabilitation, Xinhua Hospital, School of Medicine, Shanghai Jiaotong University, 1665 Kongjiang Road, Shanghai 200092, China

***Email:*** [duqing@xinhuamed.com.cn](mailto:duqing@xinhuamed.com.cn)

***Name:*** Xuan Zhou

***Address:*** Department of Rehabilitation, Xinhua Hospital, School of Medicine, Shanghai Jiaotong University, 1665 Kongjiang Road, Shanghai 200092, China

***Email:*** [zhouxuan@xinhuamed.com.cn](mailto:zhouxuan@xinhuamed.com.cn)

**Word count of the text:** 5627 (with references)

**Number of figures:** 7

**Number of tables:** 3

**PROSPERO registration number:** CRD42022352991 (Available at <http://www.crd.york.ac.uk/prospero/)>

**List of abbreviations**

| BARS | Body Awareness Rating Scale |
| --- | --- |
| BDI | Beck Depression Inventory |
| CBT | cognitive behavioral therapy |
| CI | confidence interval |
| CINAHL | Cumulative Index of Nursing and Allied Health Literature |
| Ex-HN-3 | Yintang |
| FIQ | Fibromyalgia Impact Questionnaire |
| GB34 | Yanglingquan |
| GRADE | Grading of Recommendations Assessment, Development, and Evaluation |
| HAM | Hamilton test score |
| LI4 | Hegu |
| LR3 | Taichong |
| PC6 | Neiguan |
| PPT | pressure pain threshold |
| PRISMA | Preferred Reporting Items for Systematic review and Meta-Analysis |
| RCT | Randomized Controlled Trial |
| SD | standard deviation |
| SF-12 | short form 12 questionnaire |
| SF-36 | short form 36 questionnaire |
| SMD | standardized mean difference |
| SNRI | serotonin-norepinephrine reuptake inhibitors |
| SP6 | Sanyinjiao |
| TCAs | tricyclic antidepressants |
| TCM | Traditional Chinese Medicine |
| TePsN | number of tender points |
| VAS | visual analogue scale |
| WMD | weighted mean difference |
